# Nitriding and Denitriding of Nanocrystalline Iron System with Bimodal Crystallite Size Distribution

**DOI:** 10.3390/ma15010143

**Published:** 2021-12-25

**Authors:** Aleksander Albrecht, Dariusz Moszyński

**Affiliations:** Department of Inorganic Chemical Technology and Environment Engineering, Faculty of Chemical Technology and Engineering, West Pomeranian University of Technology in Szczecin, Piastów Ave. 42, 71-065 Szczecin, Poland; dmoszynski@zut.edu.pl

**Keywords:** iron nitrides, bimodal size distribution, nanocrystalline materials, phase transformations, Gibbs–Thomson effect

## Abstract

An artificially prepared nanocrystalline iron sample with bimodal crystallite size distribution was nitrided and denitrided in the NH_3_/H_2_ atmosphere at 350 °C and 400 °C. The sample was a 1:1 mass ratio mixture of two iron samples with mean crystallite sizes of 48 nm and 21 nm. Phase transformations between α-Fe, γ’-Fe_4_N and ε-Fe_3-2_N were observed by the in situ X-ray powder diffraction method. At selected steps of nitriding or denitriding, phase transformations paused at 50% of mass conversion and resumed after prominent variation of the nitriding atmosphere. This effect was attributed to the separation of phase transformations occurring between sets of iron crystallites of 48 nm and 21 nm, respectively. This was due to the Gibbs–Thomson effect, which establishes the dependence of phase transformation conditions on crystallite sizes.

## 1. Introduction

Thermodynamic and kinetic relations concerning reactions of iron leading to the formation of iron nitrides have been developed theoretically and confirmed experimentally [1,2,3]. On this basis, a detailed phase diagram of the iron–nitrogen system has been elaborated [4,5]. The homogeneity ranges of the specific crystallographic phases occurring in this system have been, for the most part, determined from experiments carried out using iron foils and powders with dimensions of micrometres [6]. However, it was shown that these homogeneity ranges determined for nanocrystalline materials are shifted in comparison with those for its coarse equivalents [7,8,9]. It was also pointed out that the nitriding potential values, at which phase transformations between α-Fe, γ’-Fe_4_N and ε-Fe_3-2_N happen, differed between processes of the iron nitriding and iron nitrides denitriding [10]. This shift between regions of phase occurrence during nitriding and denitriding processes was described as the hysteresis phenomenon in the nanocrystalline Fe–N system. It can be rephrased as a state where different ammonia concentrations are required to form materials with identical crystallographic structures, depending on whether they were obtained by the nitriding or denitriding process.

The specific properties in nanocrystalline samples can be explained by the Gibbs–Thomson effect, for which the surface energy plays a significant role in the overall energy balance [11]. This effect manifests itself as a dependence of certain parameters on the crystallite size of the material. Among these parameters, the temperature of phase transformations [12] or the concentration of a specific constituent required to saturate a given crystallographic phase [13,14] are often reported.

In reality, powdered materials usually exhibit a certain distribution of crystallite sizes. Therefore, when determining the boundaries of parameters where given crystallographic phases occur, the influence of the crystallite size distribution should be taken into account [15]. This relation has been studied in various ways in the Fe–N system for the nitriding and denitriding processes carried out in an atmosphere containing ammonia [16,17]. In an earlier publication [17], the values of the nitriding potential required to induce the α-Fe to γ’-Fe_4_N phase transformation were analysed for iron-based materials with various mean crystallite sizes. The process was carried out in isothermal conditions. The original distribution of crystallite sizes was altered experimentally by sintering the sample at elevated temperatures. As a result of this procedure, as expected by the consideration of the Gibbs–Thomson effect, an apparent shift in the homogeneity ranges of α-Fe and γ’-Fe_4_N phases was noted. However, typical nanocrystalline iron samples have a log-normal crystallite size distribution [18]. Due to this type of size distribution, the observed changes in the Fe–N phase system were indistinct.

Thus, the hypothesis was put forward that in a material with a much more diverse crystallite size distribution, the effect of the composition of the nitriding atmosphere on the observed phase transformations would be much more distinct and observed readily. Additionally, the influence of the Gibbs–Thomson phenomenon would reveal itself much more explicitly. Ideally, a mixture would be produced from two materials with very narrow crystallite size distributions, for which the average crystallite size differs significantly. Such a material with a bimodal (or more generally multi-modal) crystallite size distribution would allow an unambiguous distinction of the thermodynamic conditions required for phase transformations to occur for crystallites of a given size. Previous studies have clearly indicated that there is a shift in the thermodynamic conditions required for the α-Fe to γ’-Fe_4_N phase transformation to occur between a material with a small value of mean crystallite size and one with a large value of mean crystallite size [17]. The idea of this work was to demonstrate that in a material that is a mixture of two components differing in the value of the mean crystallite size, it is possible to experimentally distinguish the ranges of the composition of the nitriding atmosphere corresponding to the areas of stability of particular crystallographic phases in specific ranges of crystallite size. Subjecting the described material to a nitriding atmosphere with a varying composition should highlight the separation of crystallographic phases that differ in their chemical and phase compositions. It is expected that for a given gas phase composition, a portion of the material (e.g., a subset of the smallest size crystallites) will occur as one crystallographic phase and the remaining portion of the material (e.g., a subset of the highest size crystallites) as another crystallographic phase.

## 2. Results and Discussion

In this paper, a synthetic mixture of two nanocrystalline iron samples (A and B) with significantly divergent mean crystallite sizes (MCS) was used. Sample A is nanocrystalline iron obtained by laser evaporation with a MCS of 48 nm, as assessed by the use of XRD. Sample B is a fused, highly porous iron that is characterised by a MCS equal to 21 nm, as evaluated by XRD. The physical mixture obtained from samples A and B is supposed to exhibit a bimodal crystallite size distribution. The evaluation of MCS for the mixed sample performed by the XRD experiment resulted in a value of 41 nm. The nitriding and subsequent denitriding processes were performed with the mixed sample, where this material was exposed to a nitriding atmosphere with a variable ammonia/hydrogen ratio. The processes were conducted under isothermal conditions. The composition of the material was examined by powder X-ray diffraction performed in situ with the use of a reaction chamber.

The initial, unprocessed sample of iron nanocrystallites is always superficially covered with a passive layer of iron oxides. This layer was reduced in the preliminary stage of the experiment under a hydrogen atmosphere at 500 °C. Preliminary nitriding tests proved that atmospheres containing no less than 50% ammonia are required for an α-Fe → γ’-Fe_4_N phase transformation to occur.

The main experiment consisted of two similar processes. Both were carried out under identical conditions, with the only exception being the reaction temperature. Each of these processes, at 350 °C and 400 °C, respectively, consisted of nitriding stages and denitriding stages. The nitriding stages were started under a 40:60 NH_3_/H_2_ mixture. The stable atmosphere composition was kept for half an hour, and then the main XRPD data was acquired. After the measurement was finished, the ammonia concentration was raised by 5%. The stabilisation, measurement and atmosphere composition change steps were repeated until pure ammonia was flowing through the chamber. Then, the process was reversed. The denitriding stages were conducted, where NH_3_ concentration was lowered stepwise by 5% after each hour of data acquisition.

**Figure 1:**
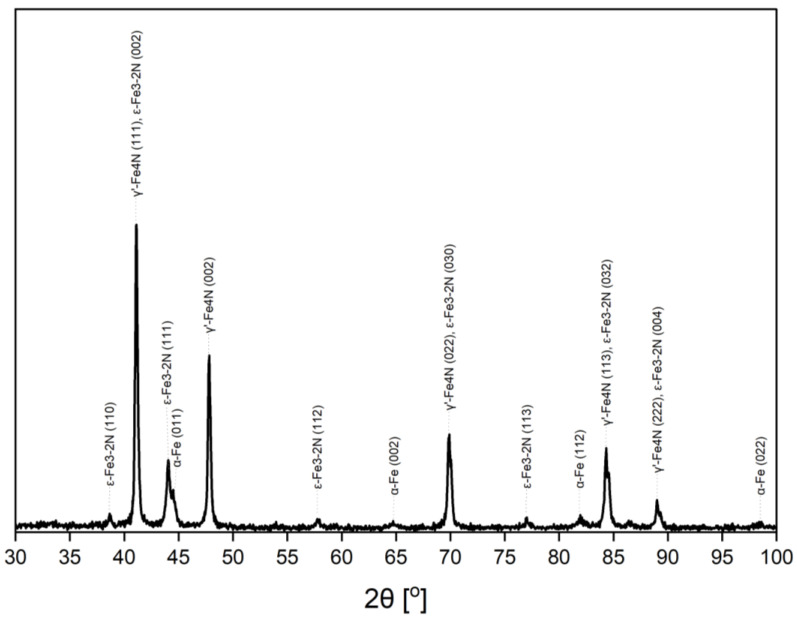
The diffractogram acquired for the gas composition of 45:55 NH_3_:H_2_ during denitriding in 350 °C, the example of the coexistence of all phases: α-Fe, γ’-Fe_4_N and ε-Fe_3-2_N identified in the examined sample.

The number of crystallographic phases, which were identified by XRPD, was different for various stages of the examined processes. However, in total, only three crystallographic phases were identified: cubic α-Fe (Im3m), cubic γ’-Fe_4_N (Fm3m) and hexagonal ε-Fe_3-2_N (P6322/P312). Phase identification was based on patterns from the ICDD PDF4 + 2020 database: 04-007-9753, 00-064-0134 and 04-007-2256, respectively. An exemplary diffractogram where all three phases coexist is shown in Figure 1. The most intensive reflections were labelled.

**Figure 2:**
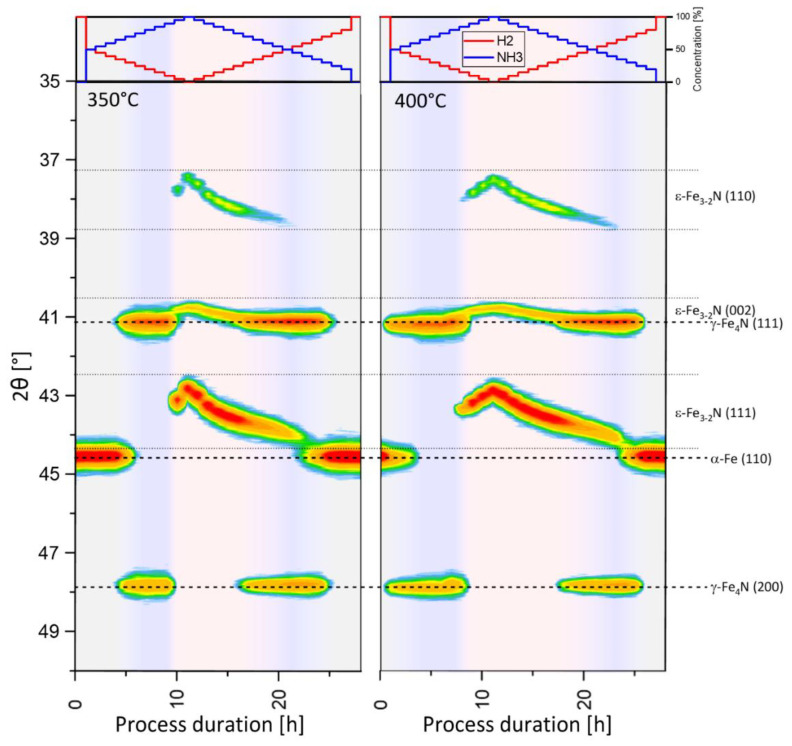
The course of the nitriding and denitriding processes performed at 350 °C and 400 °C observed by XRPD.

Combined diffraction results acquired during nitriding and denitriding stages performed at 350 °C and 400 °C are shown together in Figure 2. In the upper panels, a stepwise change in the composition of the nitriding atmosphere is depicted. The main part of the figure consists of an isoline plot. It represents the intensities of the observed reflections as a function of the 2θ angle in a selected diffraction angle range (35–50°2θ). Background colours correspond to the homogeneity ranges of α-Fe (grey), γ’-Fe_4_N (blue) and ε-Fe_3-2_N (pink) phases. Colour gradients were used to indicate regions where phases coexist.

In the beginning, with no difference for both process temperatures, only one reflection located at 44.54°2θ is observed and ascribed to the α-Fe (111) plane. This confirms that pure iron is a starting material in this process. At a temperature of 350 °C, when the ammonia concentration reaches 65%, the onset of two additional reflections at 41.16°2θ and 47.77°2θ is noticed. These reflections correspond to γ’-Fe_4_N (111) and γ’-Fe_4_N (200) planes, respectively. Starting from this stage, a mixture of α-Fe and γ’-Fe_4_N is present in the solid. Next, the α-Fe (111) reflection starts to disappear, and in the process stage, when the ammonia concentration is between 80–90%, only reflections corresponding to the γ’-Fe_4_N phase are detected. In a single measurement point, under an atmosphere containing 95% of NH_3_, the diffraction pattern is a combination of reflections coming from γ’-Fe_4_N and three other reflections located at 37.74°2θ, 40.80°2θ and 43.11°2θ that are attributed to ε-Fe_3-2_N (110), (002) and (111) planes, respectively. When pure ammonia passes through the reaction chamber, only reflections coming from the ε-Fe_3-2_N phase are observed. At that stage, the nitriding process is reversed. Further on, the ammonia content in the gas atmosphere is decreased stepwise by the addition of the corresponding amount of hydrogen. A very characteristic region of the process is observed henceforth. A gradual and monotonic shift of the reflections ε-Fe_3-2_N (110) and ε-Fe_3-2_N (111) is noted. A slightly less pronounced bend of the ε-Fe_3-2_N (002) reflection is also visible. The observed shift is directed towards higher values of diffraction angle and indicates the shrinkage of the ε-Fe_3-2_N phase lattice. It results from the removal of the nitrogen atoms from the ε-Fe_3-2_N phase induced by the decrease of the ammonia concentration in the nitriding gas [10,19]. The onset of reflections originating from γ’-Fe_4_N (111) and γ’-Fe_4_N (200) planes occurs at an ammonia concentration of 75%. At this stage, the state of the solid sample can be considered analogous to the state observed during the nitriding process just before the γ’-Fe_4_N phase vanished. However, it must be emphasised that the ammonia concentration observed in this state during the denitriding stage is much lower than the one observed for the nitriding stage (namely 95%). It evidently proves that in the nanocrystalline Fe–N system of interest, the nitriding and denitriding stages are not symmetric in the domain of gas composition. An important proof for this disparity is the succeeding region observed with a further drop in the ammonia concentration. The coexistence of γ’-Fe_4_N and ε-Fe_3-2_N phases is observed until the ammonia concentration drops to 40%, while during nitriding, this state was observed only for a single point at 95% ammonia in gas. A transitional region exists where three phases, ε-Fe_3-2_N, γ’-Fe_4_N and α-Fe, coexist followed by a region where only reflections coming from γ’-Fe_4_N and α-Fe phases are visible. Finally, below an ammonia concentration of 25%, only reflections originating from the α-Fe (111) plane are detectable.

Based on the XRD analysis of the process carried out at 350 °C, a whole nitriding–denitriding process can be divided into nine distinctive regions, which successively follow each other. The entire process begins with the α-Fe only region, followed by the transformation region with α-Fe and γ’-Fe_4_N mixtures. Next, the γ’-Fe_4_N phase region exists. With the further rise of ammonia concentration, a narrow region of ε-Fe_3-2_N and γ’-Fe_4_N coexistence occurs. At the highest concentration of ammonia, only ε-Fe_3-2_N is present in the material. After inversion of the process, from nitriding to denitriding, the nitrogen content in ε-Fe_3-2_N is depleted until the region of ε-Fe_3-2_N and γ’-Fe_4_N coexistence occurs again. Then, a coexistence of ε-Fe_3-2_N, γ’-Fe_4_N and α-Fe is observed in a narrow range of ammonia concentrations. The two final regions consist of a γ’-Fe_4_N and α-Fe mixture and pure α-Fe, respectively.

In general, the process sequence carried out at 400 °C is a reproduction of the process at 350 °C. Nine regions of phase occurrence are distinguished. There are only variances in the boundaries of the ammonia concentration where these regions are observed. The homogeneity ranges of the γ’-Fe_4_N during the nitriding stage, as well as the whole homogeneity range of the ε-Fe_3-2_N, are much broader at 400 °C. This is consistent with the potential phase diagrams constructed for the Fe–N system [4]. In these diagrams, the homogeneity range of γ’-Fe_4_N is broader at 400 °C than at 350 °C. Correspondingly, at 400 °C, the ε-Fe_3-2_N phase can exist at a lower ammonia concentration. These observations are also in accordance with the hysteresis phenomenon mentioned earlier [10].

**Figure 3:**
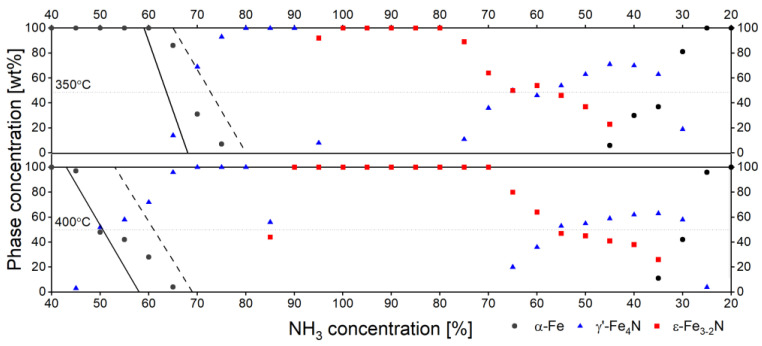
The evolution of α-Fe, γ’-Fe_4_N and ε-Fe_3-2_N phase composition of the sample during nitriding and denitriding processes studied at 350 and 400 °C. Solid (sample A) and dashed (sample B) black lines show the α-Fe concentration during α-Fe to γ’-Fe_4_N phase transformation for the similar processes carried for sample A and B separately.

Direct analysis of diffraction patterns cannot give much information on the influence of crystallite size distribution on the process of nitriding and denitriding. However, a quantitative study of the transformation of the solid during this process should give more insight into this subject. Therefore, the results presented in Figure 2 were analysed quantitatively with the use of the Rietveld method to evaluate the concentrations of all identified crystallographic phases. The results of this analysis are plotted in Figure 3.

The studied sample is assumed to have a bimodal size distribution with two distinguished maxima, at 21 nm and 48 nm. As stated earlier, it is supposed that each of these two sets of crystallites should be subject to phase transformation under somewhat different thermodynamic conditions. The parameter which is considered as decisive is ammonia concentration (precisely nitriding potential). Specifically, the smaller the mean crystallite size, the higher the ammonia concentration required to initiate the phase transformation [17]. In the case of the phase transformation α-Fe → γ’-Fe_4_N, this assumption was also checked separately for samples A and B. This phase transformation occurs for sample A (cf. solid line in Figure 3), considered to consist of crystallites of higher sizes at lower ammonia concentrations than the same phase transformation observed for sample B (cf. dashed line in Figure 3), which is considered to be a set of smaller iron crystallites. The material being the main subject of research presented in this paper is a mixture of samples A and B. From the assumptions presented at the beginning of this paper, it follows that the phase transformation for such a mixed material should occur between the lines shown in Figure 3. For both temperatures, this is actually the case. Moreover, some effects showing that about half of sample mass is converted into a particular phase and the other half remains mostly unprocessed are expected.

The state that is the most apparent candidate for being evidence of the above assumptions is the plateau visible between 65 and 55 vol% of NH_3_ for the denitriding process performed at 350 °C. This state is a result of the ε-Fe_3-2_N → γ’-Fe_4_N transformation. The transformation starts as soon as the ammonia concentration drops below 80%. However, the process is paused when exactly 50% of the mass of the sample is converted into γ’-Fe_4_N. The further decomposition of ε-Fe_3-2_N proceeds once the ammonia concentration falls below 55%.

At 400 °C, the course of the process observed in two other regions may be considered as further, though less evident, examples. During the initial stages of nitriding at 400 °C, an inflexion is observed in the region of ammonia concentration between 50 and 55%. Again, this inflexion is located almost exactly at 50% of the sample mass. It is supposed that the phase transformation α-Fe → γ’-Fe_4_N is finished at 50% of ammonia for one set of crystallites, and the other set of crystallites requires ammonia concentration in excess of 55%. The other region is analogous to the one indicated at 350 °C. It is a part of the denitriding stage, between 55% and approx. 40% of NH_3_ in gas. In the latter case, the sample mass is not explicitly fixed at 50% but varies by only 10% over the entire considered ammonia concentration range.

The three given examples indicate the possibility that there may be regions of nitriding or denitriding reactions in which some of the material has already undergone a phase transformation, and the remaining material still retains its initial form. Such a distinction in the behaviour of specific sets of material crystallites was not observable for a typical nanocrystalline sample exhibiting a log-norm distribution with a single maximum crystallite size [17]. We believe that the provided evidence is sufficient to support the hypothesis presented at the beginning, indicating the influence of the Gibbs–Thomson effect in the nitriding and denitriding process in the Fe–N system.

Nevertheless, to better distinguish the areas indicated above, the applied step of ammonia concentration increment of 5% is rather too large. It seems that an increase in resolution to 1–2% ammonia concentration increment per step is required. However, such a procedure requires a different measurement system and is planned to be performed in the future.

The studies of nitriding and denitriding of nanocrystalline Fe–N systems in variable gas atmospheres corroborate the influence of crystallite size on the concentration of ammonia required to induce phase transformations. The previously shown phenomenon of hysteresis in phase concentration to nitriding potential dependence was confirmed. An unusual reaction course was observed due to the use of two starting materials with different mean crystallite sizes, which can be explained based on the Gibbs–Thomson effect.

## 3. Materials and Methods

Sample A is commercial nanocrystalline iron obtained by laser evaporation (Sigma-Aldrich, St. Louis, MO, USA). Based on the XRD experiment, its calculated MCS is 48 nm, while that assessed by use of transmission electron microscopy is 39 nm with a standard deviation of 26 nm. Sample B is a fused, highly porous iron that is characterised by a MCS equal to 21 nm as evaluated by X-ray diffraction. An evaluation of crystallite size distribution obtained by chemical methods for identical sample results in values of 21 nm and 7 nm for MCS and standard deviation, respectively. The physical mixture obtained from samples A and B in a 1:1 mass ratio is supposed to exhibit a bimodal crystallite size distribution. The evaluation of MCS for this mixed sample performed upon XRD experiment resulted in 41 nm as a mean value.

The sample was then exposed to ammonia (UHP, Air Liquide Polska, Kraków, Poland) and hydrogen (99.999%, Messer Polska, Chorzów, Poland) mixtures of a total flow of 100 mL/min, with varying ratios controlled by mass flow regulators (Bronkhorst, Ruulo, The Netherlands). The process was performed in the reaction chamber (XRK 900, Anton Paar, Graz, Austria) mounted on the X-ray diffractometer (Philips X’Pert Pro MPD, Philips PANalytical, Almelo, The Netherlands). The chamber was equipped with a rigid ceramic holder (Macor) without built-in thermal expansion correction. The gas feed system and the reactor are designed in such a way that the chamber can be treated as a continuous, ideally stirred tank reactor. The temperature calibration was done with the phase transformation method.

In-situ X-ray powder diffraction (XRPD) analyses were conducted isothermally at temperatures of 350 °C and 400 °C, in range of diffraction angle 30–100°2θ with 165 s per step. The apparatus working in Bragg–Brentano geometry with a copper radiation source was used. To avoid fluorescence effects, a graphite monochromator was mounted on the diffracted beam. The phase analysis was performed with the use of the ICDD PDF 4 + 2020 database. A full-pattern fit based on the Rietveld method was conducted to calculate the weight fractions of the crystallographic phases identified in the material. The mean crystallite size was determined as a part of the Rietveld refinement procedure. The dependence of Full Width at Half Maximum on 2θ was fitted according to the Caglioti function, taking into account appropriate constraints. Correction of the values of the coefficients was done using the standard (powder silicon). A procedure of Rietveld refinement procedure included in the HighScore Plus 3.0 software by PANalytical was utilised.

## Data Availability

Not applicable.

## References

[1] Mittemeijer E.J., Somers M.A. (1997). Thermodynamics, kinetics, and process control of nitriding. Surf. Eng..

[2] Kooi B.J., Somers A.J., Mittemeijer E.J. (1996). An Evaluation of the Fe-N Phase Diagram Considering Long-Range Order of N Atoms in γ’-Fe_4_N_1-x_ and ε-Fe_2_N_1-z_. Metall. Mater. Trans. A.

[3] Kunze J. (1990). Nitrogen and Carbon in Iron and Steel Thermodynamics.

[4] Göhring H., Fabrichnaya O., Leineweber A., Mittemeijer E.J. (2016). Thermodynamics of the Fe-N and Fe-N-C Systems: The Fe-N and Fe-N-C Phase Diagrams Revisited. Metall. Mater. Trans. A.

[5] Wriedt H.A., Gokcen N.A., Nafziger R.H. (1990). The Fe-N (Iron-Nitrogen) System. Binary Alloy Phase Diagrams.

[6] Malinov S., Böttger A.J., Mittemeijer E.J., Pekelharing M.I., Somers M.A. (2001). Phase Transformations and Phase Equilibira in the Fe-N System at Temperatures below 573 K. Metall. Mater. Trans. A.

[7] Wohlschlögel M., Welzel U., Mittemeijer E.J. (2007). Unexpected formation of ε iron nitride by gas nitriding of nanocrystalline α-Fe films. Appl. Phys. Lett..

[8] Moszyński D. (2014). Nitriding of Nanocrystalline Iron in the Atmospheres with Variable Nitriding Potential. J. Phys. Chem. C.

[9] Pelka R., Arabczyk W. (2009). Studies of the Kinetics of Reaction between Iron Catalysts and Ammonia—Nitriding of Nanocrystalline Iron with Parallel Catalytic Ammonia Decomposition. Top. Catal..

[10] Moszyński D., Moszyńska I., Arabczyk W. (2012). Iron nitriding and reduction of iron Fenitrides in nanocrystalline -N system. Mater. Lett..

[11] Moszyński D., Moszyńska I., Arabczyk W. (2013). The transformation of α-Fe into γ’-Fe_4_N in nanocrystalline Fe-N system: Influence of Gibbs-Thomson effect. Appl. Phys. Lett..

[12] Mayo M.J., Suresh A., Porter W.D. (2003). Thermodynamics for nanosystems: Grain and particle-size dependent phase diagrams. Rev. Adv. Mater. Sci..

[13] Perez M. (2005). Gibbs–Thomson effects in phase transformations. Scripta Mater..

[14] Mukherjee R., Choudhury A., Nestler B. (2013). Composition pathway in Fe–Cu–Ni alloy during coarsening. Modell. Simul. Mater. Sci. Eng..

[15] Pelka R., Arabczyk W. (2013). A New Method for Determining the Nanocrystallite Size Distribution in Systems Where Chemical Reaction between Solid and a Gas Phase Occurs. J. Nanomater..

[16] Wilk B., Pelka R., Arabczyk W. (2017). Study of the Iron Catalyst for Ammonia Synthesis by Chemical Potential Programmed Reaction Method. J. Phys. Chem. C.

[17] Moszyński D., Kiełbasa K., Arabczyk W. (2013). Influence of crystallites’ size on iron nitriding and reduction of iron nitrides in nanocrystalline Fe-N system. Mater. Chem. Phys..

[18] Pielaszek R. (2004). FW15/45M method for determination of the grain size distribution from powder diffraction line profile. J. Alloys Compd..

[19] Somers MA J., Kooi B.J., Maldzinski L., Mittemeijer E.J., van der Horst A.A., van der Kraan A.M., van der Pers N.M. (1997). Thermodynamics and long-range order of the interstitials in an h.c.p lattice: Nitrogen in ε-Fe_2_N_1-z_. Acta Mater..

